# Perspectives and Challenges of Microbial Application for Crop Improvement

**DOI:** 10.3389/fpls.2017.00049

**Published:** 2017-02-09

**Authors:** Salme Timmusk, Lawrence Behers, Julia Muthoni, Anthony Muraya, Anne-Charlotte Aronsson

**Affiliations:** ^1^Department of Forest Mycology and Plant Pathology, Uppsala BioCenter, SLUUppsala, Sweden; ^2^Nova West Technologies and CommunicationsTucson, AZ, USA; ^3^SLU Holding ABUppsala, Sweden

**Keywords:** plant growth promoting rhizobacteria (PGPR), biofertilizers, biopecticides, commercial application, systems biology

## Abstract

Global population increases and climate change pose a challenge to worldwide crop production. There is a need to intensify agricultural production in a sustainable manner and to find solutions to combat abiotic stress, pathogens, and pests. Plants are associated with complex microbiomes, which have an ability to promote plant growth and stress tolerance, support plant nutrition, and antagonize plant pathogens. The integration of beneficial plant-microbe and microbiome interactions may represent a promising sustainable solution to improve agricultural production. The widespread commercial use of the plant beneficial microorganisms will require a number of issues addressed. Systems approach using microscale information technology for microbiome metabolic reconstruction has potential to advance the microbial reproducible application under natural conditions.

## Introduction

According to the Food and Agriculture Organization (FAO), the estimated world population for 2025 will be nearly 8.5 × 10^9^ inhabitants. Such an increase will inevitably require substantial additional agricultural production of ~2.4 × 10^9^ t/year. At the same time agriculture faces several unexpected environmental challenges that are particularly acute in low income countries as agriculture represents the dominant part of their economy. Food and water shortages can lead to further unrest and wars. It is generally accepted that the above mentioned increase in agricultural production should not be based on an increase in the arable surface but rather on increased production on existing agricultural land via improvement of crop productivity. In this context, the use of microbial inoculants is one potential way of realizing this goal. In fact, this approach has recently gained popularity and a number of new products have been formulated. Application of the products has been recently elaborately reviewed and product lists published (Calvo et al., 2014; Pertot et al., 2016; Singh et al., 2016).

## Definitions

In natural conditions, both plant above ground organs and its rhizosphere are colonized by bacteria, fungi, actinomycetes, protozoa, and algae. Ninety five percent of all the colonizing microorganisms are bacteria (Glick, 2012). Plant Growth Promoting Bacteria (PGPB) or Rhizobacteria (PGPR), as on the majority of cases the effect is caused by the bacteria living on or inside plant roots, are defined as the bacteria that exert highly beneficial effects on plant development by direct or indirect mechanisms. Some PGPB/PGPR can be classified as biofertilizers and biocontrol agents or biopesticides (Glick, 2012). Biofertilizers are the rhizobacteria that under particular conditions mainly enhance plant growth via providing required nutrition. The bacteria can accelerate certain microbial processes in the soil that augment the availability of nutrients in a form easy to assimilate by plants. They can be grouped, based on their nature and function, as N_2_ fixing, phosphate solubilizing, phosphate mobilizing, or biofertilizers for micronutrients. Biocontrol agents (BCA) or biopesticides main function is suppressing or controlling plant disease. They can be categorized as bacteria *per se* or as compounds derived from bacteria acting as biocontrol agents. Most likely plants use different strategies for growth promotion at different times of plant growth and development (Glick, 2012). All these mechanisms of plant growth promotion act dynamically, and only by monitoring the various parameters over the entire growth period, we can estimate the role of each of the different factors.

## General overview of PGPB/PGPRs

**Figure 1:**
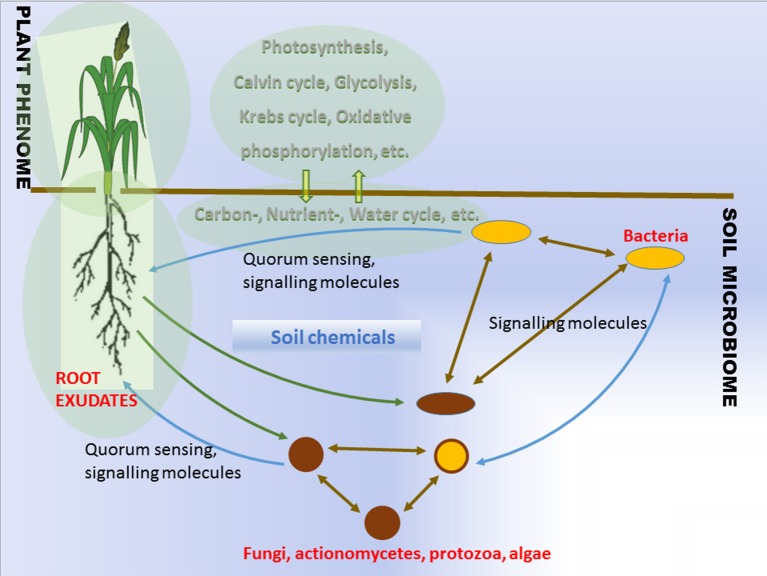
**Schematic overview of interactions between plant and soil microbiome**.

The soil that surrounds plant roots is a primary source of the bacterial agents promoting plant growth. Plants influence the proliferation of soil microorganisms in the vicinity of their roots via root exudation (Walker et al., 2003). The rhizosphere effect was already known in the beginning of the twentieth century (Hiltner, 1904). Plant genotype, health, developmental stage, and fitness determine the composition and properties of the root exudates. Root exudation together with mucilage, lost cap-, border-, epidermal and cortical cells, and with soil chemicals, leads to the changes in pH and redox gradients which help to shape the microbial communities around roots (Figure 1, Lareen et al., 2016). Understanding the principals of microbe-microbe and plant-microbe communication provides the potential to generate beneficial microbial communities in agricultural soils. The question is whether this is feasible and whether such microbial communities would be stable enough to function reproducibly under agricultural conditions.

Notwithstanding the differences between PGPB/PGPR, they all utilize similar mechanisms. Namely PGPB/PGPR promote plant growth directly or indirectly (Glick, 1995, 2012). This occurs as the result of providing plants with particular compounds or by lessening the severity of disease. These particular mechanisms are not always well understood but the possible explanations for the direct promotion of plant growth include: hormones produced such as, abscisic acid, gibberellic acid, cytokinins, and auxins; important enzymes like, 1-aminocyclopropane- 1-carboxylate (ACC) deaminase to decrease the amount of ethylene in emerging plants and plants under stress conditions; contributing systemic resistance by compounds produced by bacteria, biofilm formation by rhizobacteria, extracellular matrix (Ryu et al., 2004; Timmusk et al., 2005, 2014; Prime-A-Plant Group et al., 2006; Timmusk and Nevo, 2011; Timmusk and Behers, 2012; Kim et al., 2013).

Commercial development of various rhizobium inoculants was initiated more than 100 years ago (Bashan, 1998). The first commercial asymbiotic PGPB/PGPR, *Bacillus thuringiensis*, was discovered as an insect pathogen, in the beginning of the 1900s. Sporeine, a compound based on *B. thuringiensis*, was the first commercial biopesticide. It was available in France in 1938. Serious industrial development of products based on *B. thuringiensis* started in the 1960s–1970s. The development and use of microbial-based fertilizers has increased throughout the world, because of the damage to the environment which is caused by extreme and unsuitable use of chemical fertilizers and by the advance of knowledge regarding the association among plants and soil microorganisms. The advancement has also stimulated work in continuing to isolate and select the best plant growth promoting capabilities by directly and or indirectly improving the plant nutrient uptake.

**Figure 2:**
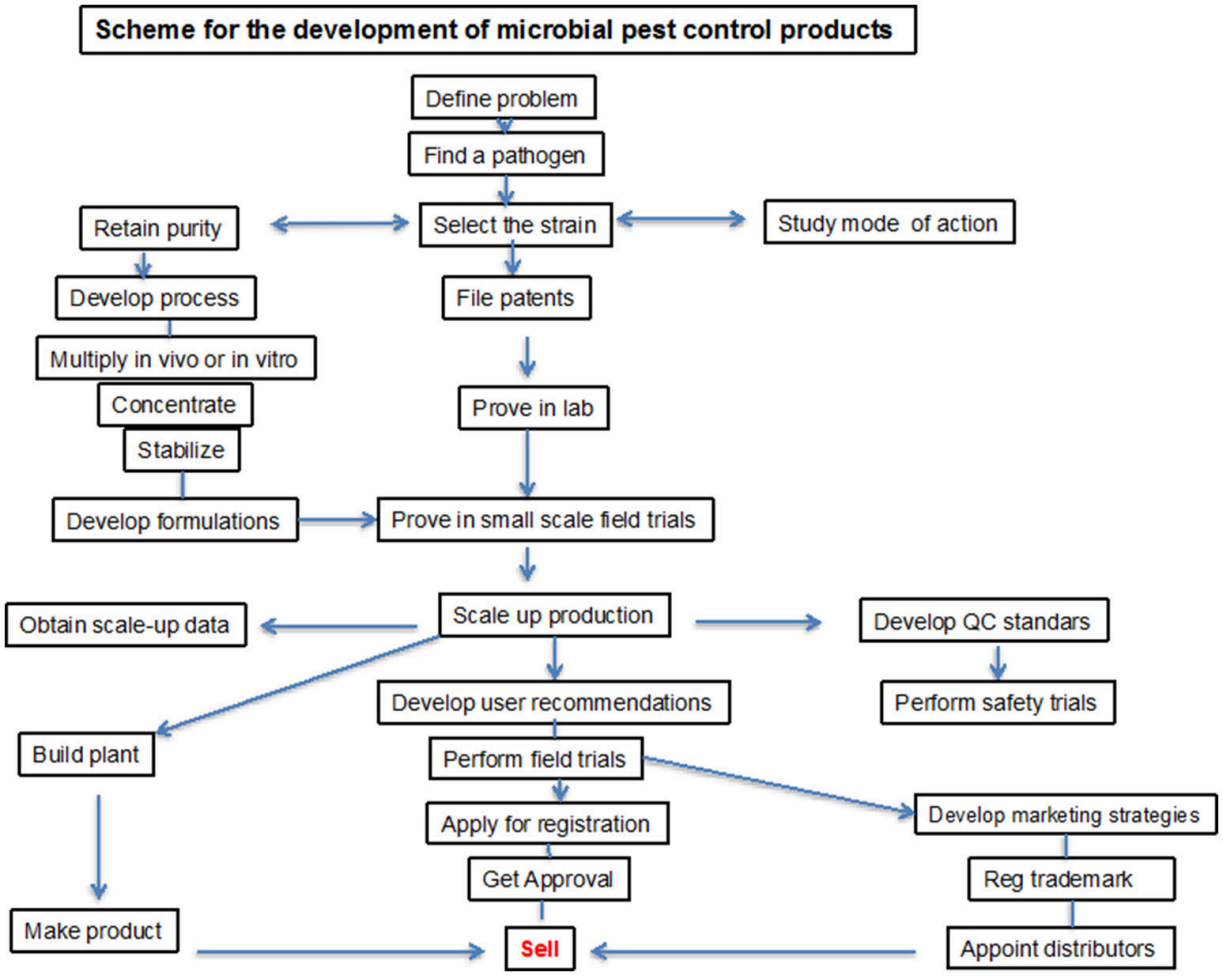
**A procedure for development of microbial product**.

Because of the positive agronomical effect of microbial-based products, a worldwide market of a new kind of PGPB/PGPR based fertilizers opened up and they have been commercially available in many countries since the 1950s. Development of commercial PGPB/PGPR product is a complex process, which requires high competence and close collaboration of specialists in various fields. The product development requires several steps addressed to isolation, screening by means of efficiency *in vitro* and *in vivo* as well as trials under natural conditions. For commercial delivery the product must be produced on a commercial scale, preserved for storage, and formulated to ensure biocompatibility. These processes may be patented for commercial use (Figure 2). However, despite a high number of patents only few have materialized in a register for agricultural application.

## The global PGPB/PGPR market

PGPB/PGPRs are addressed commercially as biofertilizers and biopesticides. Hence the terminology is used in the chapter.

**Figure 3:**
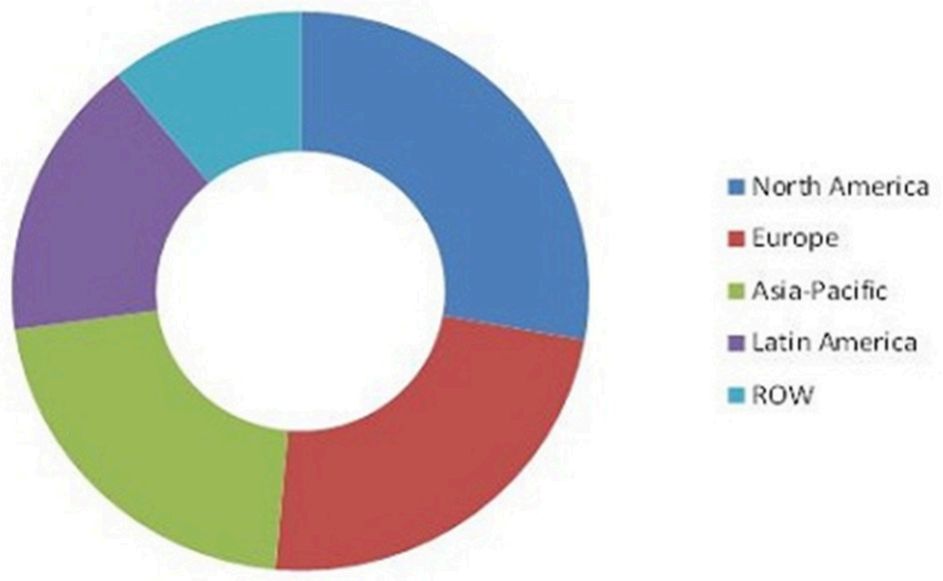
**Worldwide market for biofertilizers by 2014 (Marketsandmarkets, 2014b)**.

The biofertilizer and biopesticide markets are segmented based on product type, active ingredients, crop type, application, and geography. Transparency Market Research has published a report on the global pesticide market value, stating it was US $1.72 billion up to 2014. It is also expected to reach US$4.17 billion by the year 2023, this is compounding the annual growth rate (CAGR) at 9.9%, between 2015 and 2023. Expectations are for North America to dominate the global biopesticide/biofertilizer market in terms of demand over the forecast period (Figure 3, Marketsandmarkets, 2014a). It is predicted that biofertilizer market share will reach USD 1.66 billion by 2022 and will rise at a CAGR of 13.2% during the years of 2015–2022.

**Figure 4:**
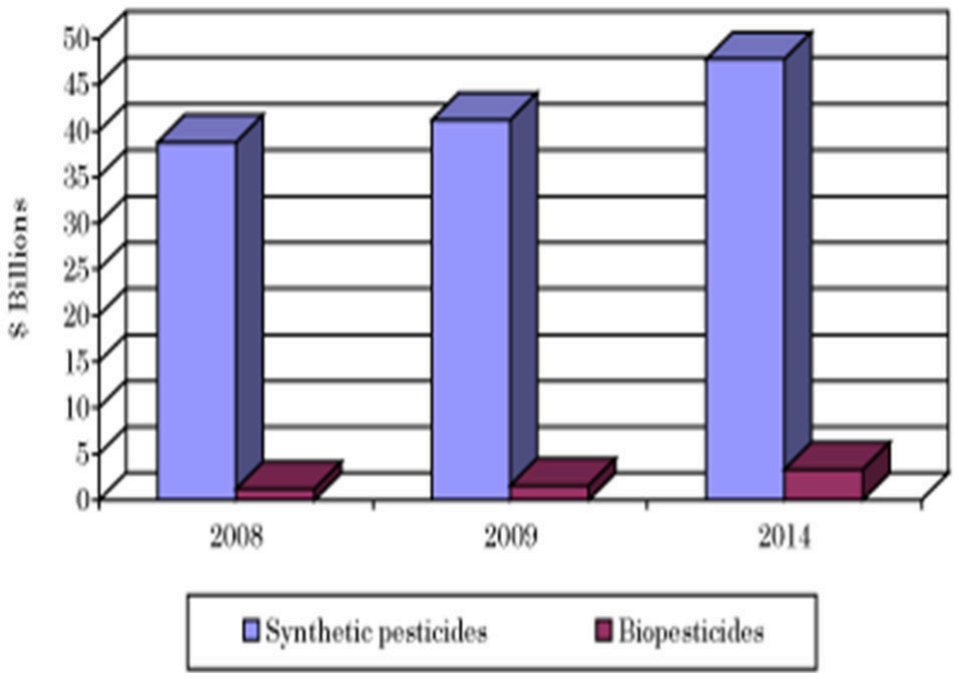
**Comparison of the market for synthetic pesticides and biopesticides (BCC Research, 2014)**.

**Figure 5:**
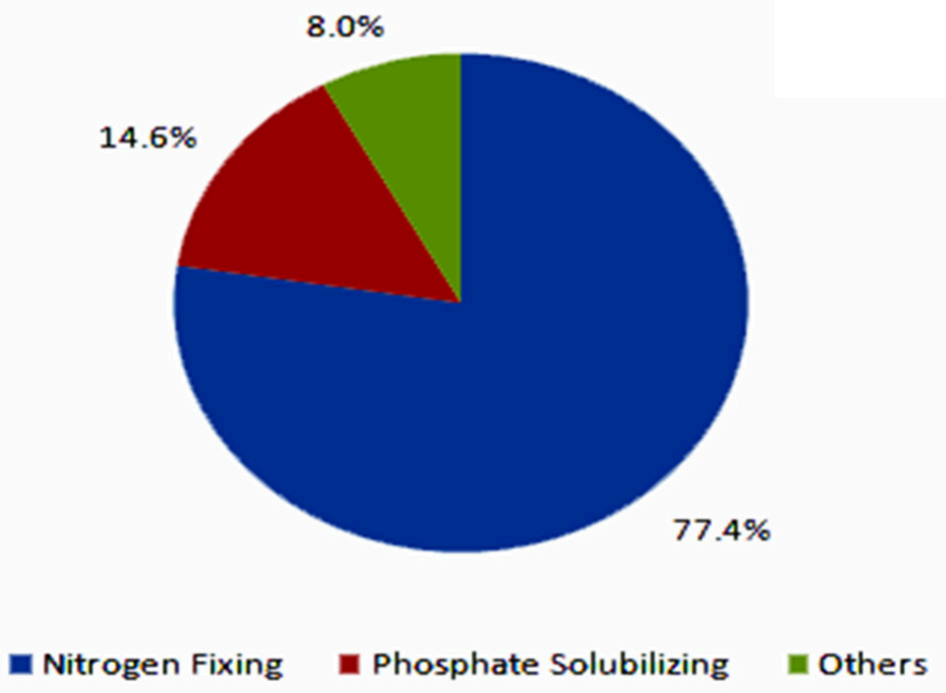
**Global biofertilizer market by product by 2012 (BCC Research, 2014)**.

The current biofertilizer market represents about 5% of the total chemical fertilizers market (Figure 4, BCC Research, 2014). The global biofertilizer market is currently dominated by nitrogen-fixing organisms as nitrogen is the most essential nutrient for plants (Figure 5). The various microorganisms used as nitrogen-supplying biofertilizers are *Rhizobium* spp., *Actinorhizobium* spp., *Azotobacter* spp., *and Azospirillum* spp. They are mainly used for leguminous crops, but the products are used to grow other crops as well, especially rice and sugarcane.

The global market for biopesticides in terms of revenues was estimated to be worth about 5 billion USD in 2011 (Marketsandmarkets, 2014a; Yaish et al., 2016) which is about 2.5% of the global market for chemical pesticides.

Geographically, the North American region had the highest demand for biofertilizers in 2013. It is projected that the region of the Asia Pacific will be the most upward growth market for biofertizers from the years 2014–2019.

### Biopesticide market in the EU by 2014^1^

The prices of microbial biopesticides are currently, at least, 25% higher than those of conventional pesticides, and this price difference is expected to expand further with the expected reduction in the prices of conventional pesticides. Ironically, even though prices of microbial biopesticides in Europe are expected to remain stable during the forecast period, actual prices are expected to increase with value addition in terms of services and new technology (Marketsandmarkets, 2014b).

### Biofertilizer market in the USA and Canada

The revenue for the North American biofertilizer market has expectations of reaching $205.6 million with a CAGR of 6.4 percent through 2011–2018. In terms of revenue, legume biofertilizers are the largest segment, accounting for 72.5% of the total for 2011 with expected growth at a CAGR around 5.3%, between 2011 and 2018. The revenue for non-legume biofertilizers is expected to grow at a CAGR of 9.2% from 2011 to 2018. The North American market share of legume biofertilizers, predominantly nitrogen-fixing Rhizobia, is estimated to have a share of about 72.5%. While the growth for legume biofertilizers in North America was high during the past years, the market is slowing down to an annual growth rate compounded at 4.5% from 2011 to 2018. The North American biofertilizer market is highly consolidated with two major players (Novozymes and Becker Underwood) together with a market of about 85.0%.

## Challenges with PGPR/PGPB product commercial applications

The large number of publications on PGPB/PGPRs demonstrates that there is growing evidence supporting the use of the products as agricultural inputs. The bacteria are being used quite effectively in many of the developing countries. Throughout the more developed world, where agricultural chemicals remain relatively inexpensive, the use of PGPR occupies a small but growing niche in the development of organic agriculture. The microbial inocula have several advantages over artificial agricultural chemicals. They are environmental friendly renewable sources of nutrients and they activate soil biology and restore soil fertility. In addition to fighting agricultural pathogens, microbes can also alleviate abiotic stresses (Timmusk and Wagner, 1999; Timmusk et al., 2014, 2015; Bharti et al., 2016; Sharma et al., 2016). However, the more widespread utilization of PGPB/PGPRs will necessitate that a number of issues be addressed.

What are the reasons, and which are the critical areas where new inputs are required in order to increase commercialization of PGPB/PGPR products? One of the main limiting more widespread use of PGPB/PGPRs is their selectivity. Conventional agrochemicals are as a rule broad-spectrum products that impact many different kind of organisms. PGPB/PGPR, on the other hand, tend to be highly targeted. This can result in variable quality and efficacy under field conditions i.e. in the complex field environment where various players act simultaneously. In addition, as detailed below, there are challenges with the microbial product registration process.

### Challenges with product registration

Regulations in the European Union with respect to the lack of quality restrictions concerning biofertilizers has left a situation where national or regional rules are applied; these are often variable and not consistent with one another.

Regulatory processes and documentation for product registration are very complex and requires significant levels of expertise. The procedure for registration of BCAs within EU is long and complicated. It is a two-phased process. The active ingredient within a biofertilizer must be authorized by the EU Commission DG SANCO (Directorate General for Health and Consumer Affairs) and subsequently, the formulated product is still a matter of national authorization. For authorization of the active substance the producer selects a Rapporteur Member State (RMS) to evaluate their dossier. The first step of the assessment by the RMS is to ensure that the application dossier (complete data package) is compliant with the requirements of the Regulation (this takes up to 6 months). If the dossier is complete RMS produces a Draft Assessment Report (DAR) that takes approximately a year. The European Food Safety Authority (EFSA) and the European Commission (EC) make comments and several expert review rounds take place (taking an additional 2–3 years). Finally the DAR is submitted to EFSA. Conclusions written by the EFSA are responded by decisions from the EC. The process can be long and take as many as several years, while deadlines are not met and numerous steps are difficult to comprehend. A number of countries respond with their own series of requirements in their specific language and additional data can be requested. Fees are high and differ considerably from one country to another. Plant growth products are being used in a number of countries and are not regulated or subjected to a level of regulation below that of biopesticides. The question is only what is defined as a pure plant-growth product in comparison to a biopesticide.

However, there is hope for an improvement. Policy in the EU concerning developing the agricultural sector, emphasizes the necessity for a reduction in the use of chemical agents with the requisite of increasing the use of substitutes to environmentally dangerous chemical agents. The EU put forward a number of legislative measures in 2009 based around Integrated Pest Management (IPM), including the Framework Directive on the Sustainable Use of Pesticides (EU DG Environment). The idea behind IPM is to combine different crop management practices to overcome the shortcomings of the individual system. IPM principles do not become mandatory until 2014, but member states have been encouraged to use rural development programs (funded under the Common Agricultural Policy) to provide financial incentives to farmers to start implementing. The new legislation gives a specific status to non-chemical and natural alternatives to conventional chemical pesticides and requires them to be given priority wherever possible. Biopesticides should generally qualify as low-risk active substances under the legislation. Low-risk substances are granted initial approval for 15 years rather than the standard 10. A reduced dossier can be submitted for low-risk substances but this has to include a demonstration of sufficient efficacy. One requirement for low-risk substances, that is still to be elaborated, is that their half-life in the soil should be less than 60 days; this may cause problems for some microbial biopesticides, such as rhizosphere-competent antagonists of soil-borne plant pathogens.

### Quality and efficacy of the products under natural conditions

It is clear that the problem of inconsistency on field application calls for innovative solutions. Soils play a privotal role in major biogeochemical cycles (carbon-, nutrient-, and water cycles) while hosting a largest microbial diversity on land (Figure 1). The ability of plant roots to produce exudates capable of attracting beneficial microorganisms is a known feature of plant metabolism. The role of root exudates is complex since it involves plants active communication with soil microorganisms, helping to attract beneficial and potentially symbiotic microorganisms (Figure 1). The beneficial effects of the microorganisms to the plants exemplified by improved stress tolerance, nutrient delivery, are influenced by diverse growth habitats and background microbial communities. The description of a microbial community, where the bacterial members are isolated, creates a problem since we currently lack sufficient knowledge in the cultivation of a high percentage of the microbes. In addition, any changes to be determined to the microbial community while it is being exposed to stressful conditions, must involve a differential quantitative technique in order to estimate on an individual basis the taxon abundance that exists within the community. Hence a major problem with PGPB/PGPR product application for crop improvement comes from the diverse growth habitat and community structure of plant roots (Figure 1).

The most fundamental objective in PGPB/PGPR application is production of properties such as biological activities to the target crop plant. This demands innovative strategies directing the product metabolism to the desired location. Understanding the dynamics of the root microbiome community and flux in plant metabolic networks (Figure 1) increases the likelihood for product effectiveness and reproducibility. In the last decade, technology has provided us with innovations and advancements and with the understanding that a crucial component to the advancement of treating with microbial products, is to differentiate between genes and the ways they initiate positive and neutral passenger actions in PGPB/PGPR and crop plant interaction. The perception that biology is an information science is already widely accepted in medical science (Edwards and Palsson, 1999; Schilling et al., 2000; Aderem and Hood, 2001; Price et al., 2002; Herrgård et al., 2008; Mo and Palsson, 2009; Bordbar et al., 2011; Palsson, 2011; Chang et al., 2013; Nam et al., 2014; Rolfsson and Palsson, 2015). Complex processes can be broken down into their component parts in a similar way as traditional engineering disciplines (Andrianantoandro et al., 2006; Pulendran et al., 2010, Figure 6). Complex metabolic processes can be converted into a mathematical format of the underlying biochemical genetic and genomic knowledge (BiGG). This format allows the formulation of genome scale models (GEMs). GEMs enable the computation of phenotypic traits of the organism or system of interest. The systems approach to immunity has been especially rewarding in vaccination science (Andrianantoandro et al., 2006; Pulendran et al., 2010, Figure 7). Information technology applied to clinical trials leads to generation of novel ideas and hypothesis and eventually results in advanced vaccine development. E.g., immune responses to vaccination in clinical trials can be profiled to depth with omics- technologies. The data are mined by bioinformatics tools and help to create hypothesis about the biological mechanisms behind the phenotype. The hypothesis is tested in human *in vitro* systems or animal models. This approach links discovery based science to clinical trials (Pulendran et al., 2010).

**Figure 6:**
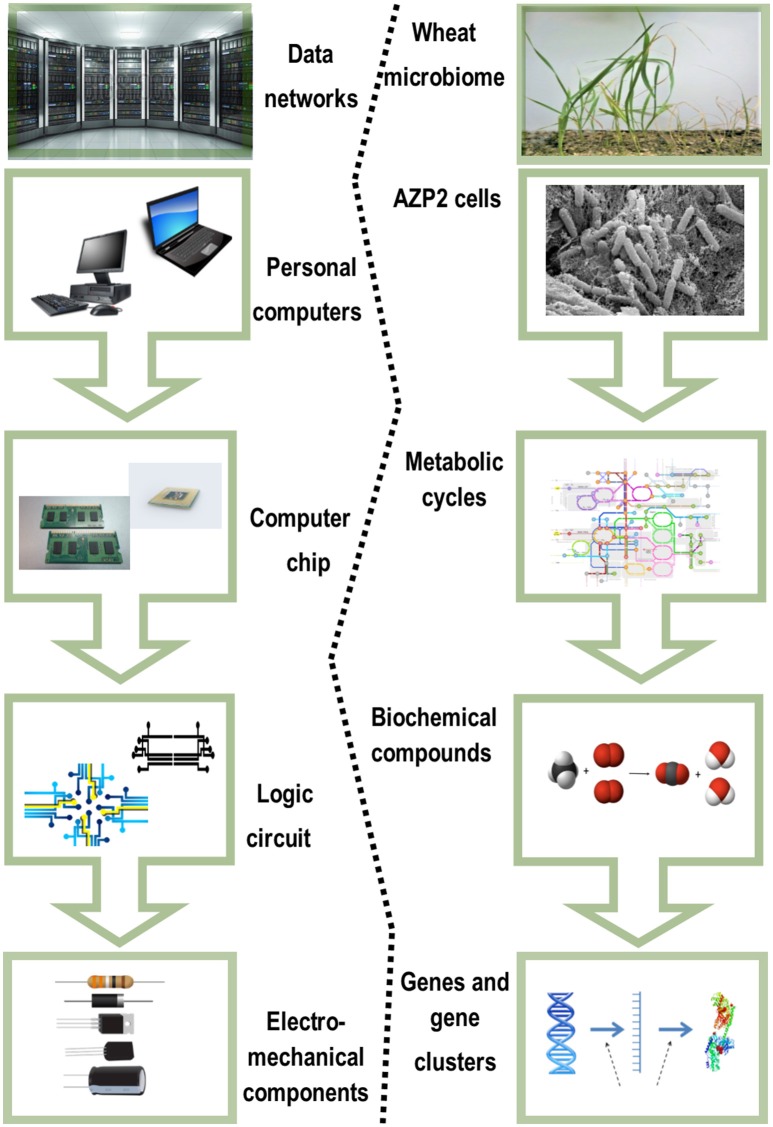
**Complex biological systems can be broken down to their component parts similar to traditional engineering disciplines**. Adapted for the *Bacillus thuringiensis* AZP2 and wheat microbiome (Timmusk et al., 2014).

**Figure 7:**
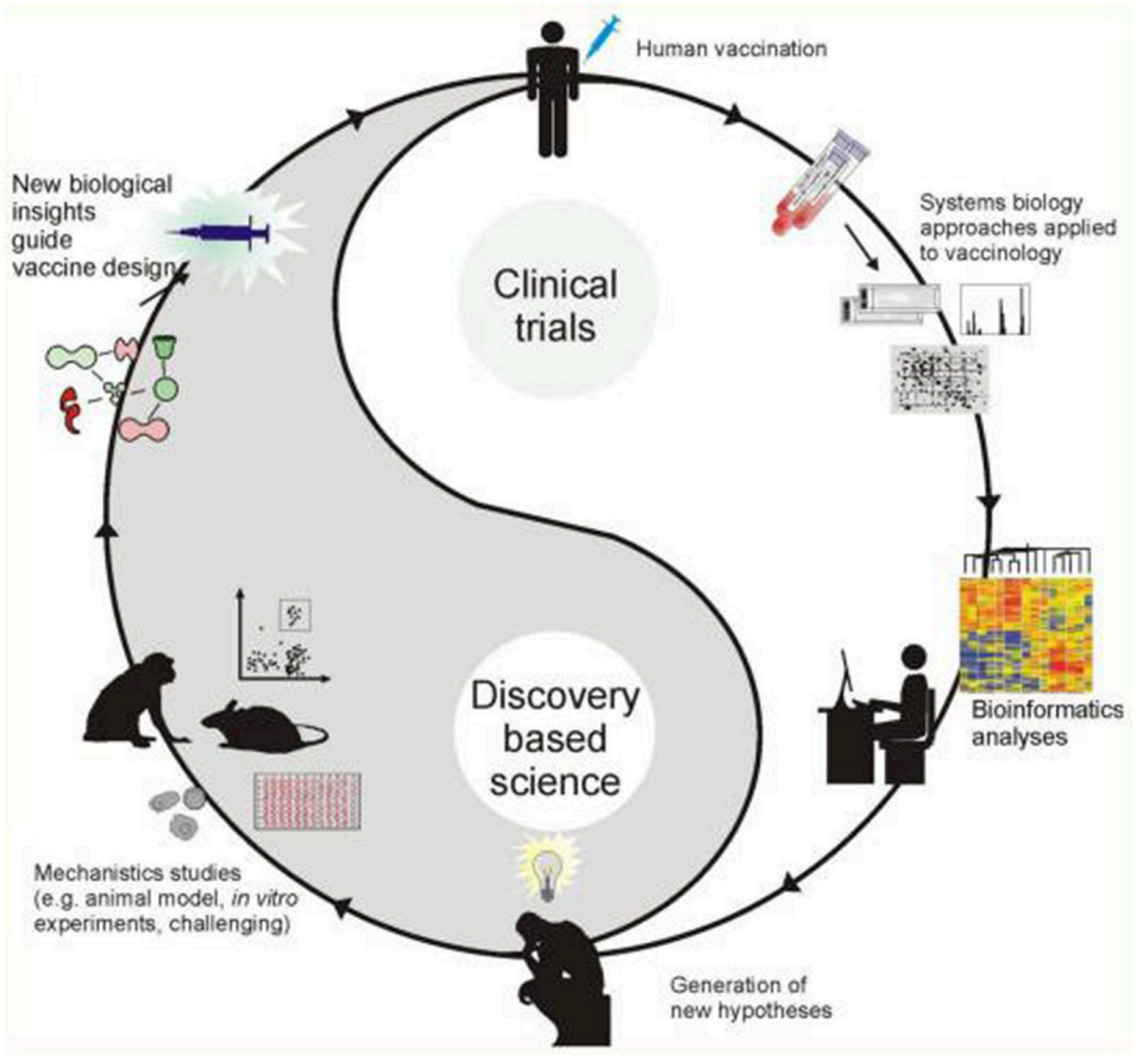
**A framework for systems vaccinology (Source Pulendran et al., 2010)**.

**Figure 8:**
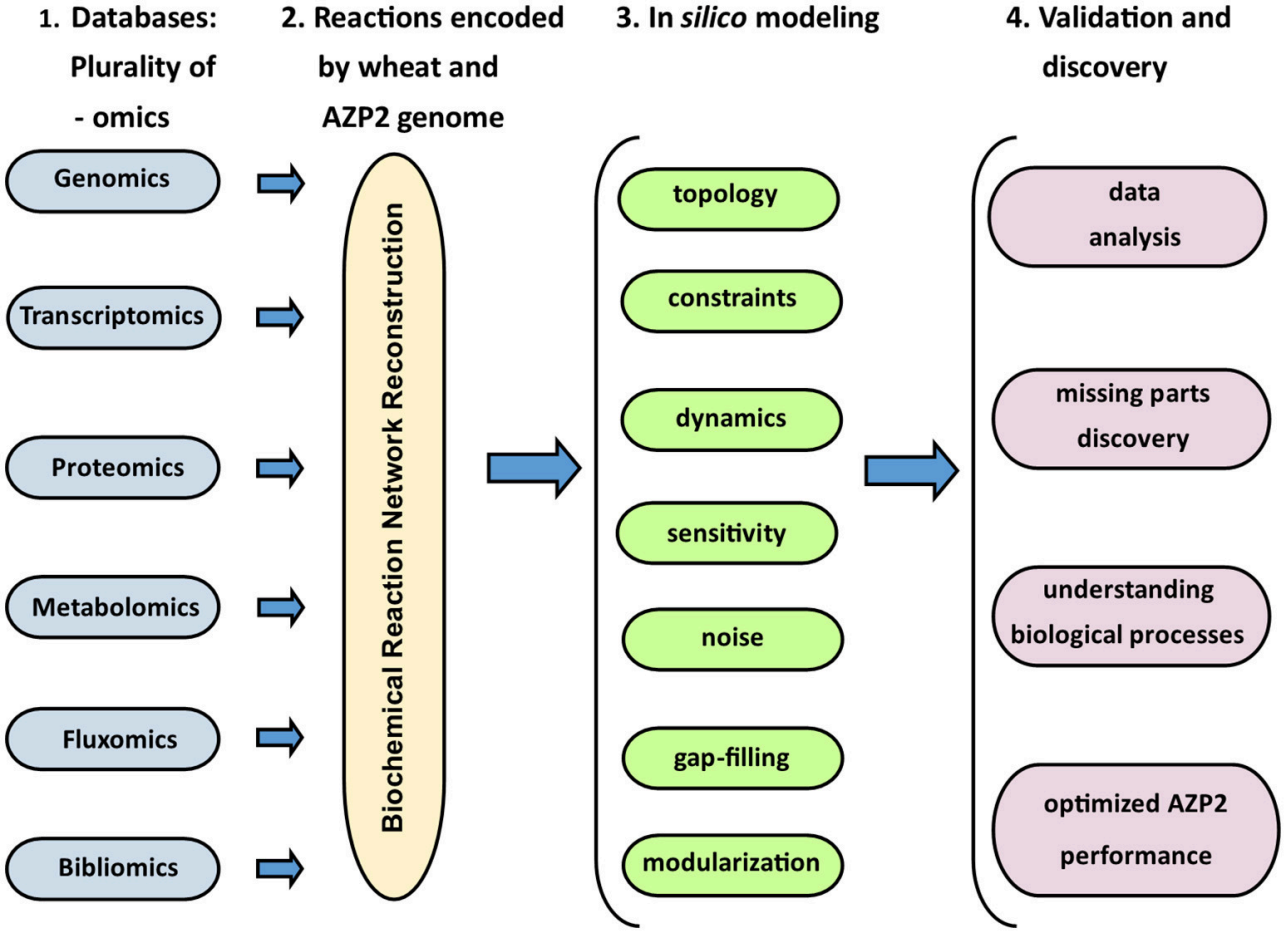
**The four-step model for metabolic systems biology**. Adapted for the *Bacillus thuringiensis* AZP2 metabolic system (Timmusk et al., 2014).

In the grand scheme of things PGPB/PGPR crop plant interaction is yet another metabolic system aside medical and other environmental systems. Since the first genome scale reconstruction 1999, the number of metabolic network reconstructions has grown exponentially, as also exponential number of plant and microbe genome sequences have been published (Edwards and Palsson, 1999). We can now estimate numerous cellular components, describe their interactions chemically, and mathematically, and as a result identify the constraints that the network operates under. This makes it possible to optimize the physiological functions in a given environment. These capabilities provide a reliable framework on which a mechanistic basis for the microbial metabolic genotype-phenotype connection can be formulated. The core process is based on an emerging archetype to relate the genotype to the phenotype through reconstruction and *in silico* model creation (Figure 8). For example, AZP2 performance in wheat microbiome is a metabolic system for *in silico* modeling (Figure 6). According to the four-step paradigm for metabolic systems biology the generation of “omics” and collection of literature data on the target organism is followed by the network reconstruction and the formulation of a BiGG knowledge base. The metabolic reconstruction is converted into a mathematical format and *in silico* query tools are implemented. This enables a variety of basic and applied uses of the reconstruction. Metabolic reconstruction- based computer simulations could be performed to evaluate e.g., microbial inoculant survival in rhizospheres. Several abiotic factors (variables) have an effect on PGPB/PGPR survival. These include the availability of soluble organic compounds and molecular oxygen, and the concentration of mineral nitrogen in soil. The principal biotic variables considered could be e.g., the direct and indirect interactions between PGPB/PGPR and resident microorganisms, protozoan predation, and bacterial parasitism (Figure 1). The problem is that in plant rhizospheres all the variables act dynamically on various levels. This kind of complexity is a challenge for factorial design that may not be easy to overcome.

Recent advances have made it possible to examine the microbial communities under natural conditions and revealed that complex microbial communities in the form of biofilms are an inevitable part of plant microbiome as a layer of protection and homeostasis (Davey and O'Toole, 2000; Timmusk et al., 2005, 2014; Timmusk, 2010, 2016; Timmusk and Nevo, 2011). The biofilms may be composed of a population that has developed from a single species or a community derived from multiple microbial species. The microbes coordinate their metabolic activity and gene expression according to the density of their local population, a process called quorum sensing (Figure 1). Remarkable discoveries have occurred in the biofilm research showing that biofilms represent systems with high level organization (van Gestel et al., 2015). Several PGPRs form large and thick bacterial clumps as a biofilm which influence plant growth via facilitating root hair length and density and improve mulch biofilm formation (Timmusk et al., 2014). The kind of stable dense biofilm matrix layer around roots is able to protect against pathogens, limit diffusion of biologically active compounds secreted by bacteria and due to smaller number of variables compare to rhizosphere bacteria is a likely option for GEM formulation.

Many bacterial endophytes have attracted considerable attention for their capacity to promote plant growth. Plant and endophytic colonization is a complex process that requires the capacity of bacteria to compete in the rhizosphere soil to find a place to communicate and interact with the plant roots. Yet endophyte based plant growth promotion is generally considered somewhat better controlled compared to the beneficial behavior of free-living soil bacteria. As an example, any variation in any number of abiotic factors, for instance, light emission, temperature, pH, soil type, and competition for nutrients, oxygen availability and the degree of interaction with other microorganisms, are vital elements that create different strategies and develop interaction among the other organisms, their existence and survivability within the plant. As a result, the possibility exists that the use of plant growth promoting bacteria lies in modeling the behavior of bacterial endophytes and biofilms.

The biological systems information pathways and networks integrated are deciphered using computer science and applied mathematics as the set of data developed is vast. Traditionally, PGPB/PGPR experimentation has been done in a one-factor-at- time manner. These methods very often yield misleading results. To increase efficiency of microbial application, ecosystem models i.e., mathematical representations of agro-ecosystems are required. Design of experiments (DoE) is a method by which purposeful changes can be made to input factors of the process in order to compare, characterize, model and optimize the effects of the output and identify significant factors which influence the inoculation process (Zhang et al., 2014). For example, the key components performance of plant metabolic cycles can be optimized considering the critical components in inoculation process (Figures 1 and 8). Factorial designs, which are a very basic type of DoE, require a minimal number of runs to identify interactions in the process of interest (Buyel and Fischer, 2014; Zhang et al., 2014; Penny et al., 2016). This information can lead to advances in procedure perception, which improves the overall quality, while decreasing the costs and seeing a beneficial increase in inoculation.

Hence, in order to enhance the consistency of PGPB/PGPR field applications we need innovative approaches picking the best existing genomic and molecular technologies available for reconstructing biochemical reaction networks (Figure 8). The networks form a base for *in silico* modeling followed by validation and understanding of biological system. In this way we should be able to create systems for the better understanding of microbial isolates and their bioactive compounds. As a result it is possible to create a map of the host plant microbiome. It is not a map of content but a map of risk in terms of probability of introduced isolates colonization, fate and efficiency. Any of the effective monitoring methods should detect plant stress and/or its alleviation in the first stages before the stress severity is detected by the visual symptoms. Early monitoring provides the means to deal with the stress situation well before the effects become irreversible and the crop yield is totally compromised. We now must create practical ways to use these maps. The aim of such maps would be trying to find an optimal fairway, inoculation time, mode and quantity i.e., a navigable channel, for maximizing the isolate's expected positive effect and minimizing the time it takes. In this way, we will be driving both science and agriculture and contributing to smart agriculture and would create a green environmental technology telling us how to properly arrange our activity concerning the isolates in an agricultural context.

## Conclusions

In order that PGPB/PGPRs may be used most effectively, there needs to be a rational approach in providing a choice and delivery of specific PGPB/PGPR directly to the field. The consideration here will depend on a series of variables. The development of mathematical models based “customized” inocula would facilitate the stable employment of PGPB/PGPR in increasing crop production. This would ensure that the great potential of PGPB/PGPR science would find its way to facilitating reproducible field application and sustainable food production under changing climate.

## Author contributions

ST, LB, JM, AM, AA contributed equally writing the review.

### Conflict of interest statement

The authors declare that the research was conducted in the absence of any commercial or financial relationships that could be construed as a potential conflict of interest.

## Footnotes

^1^Research, 2016 G. V. Market and Consulting: Biofertilizers Market Analysis by Product and Segment Forecasts to 2020.
